# First Report on Molecular Characterization of *Taenia multiceps* Isolates From Sheep and Goats in Faisalabad, Pakistan

**DOI:** 10.3389/fvets.2020.594599

**Published:** 2020-11-10

**Authors:** Mughees Aizaz Alvi, John Asekhaen Ohiolei, Muhammad Saqib, Muhammad Haleem Tayyab, Muhammad Umar Zafar Khan, Li Li, Amjad Islam Aqib, Ali Hassan, Anum Aizaz Alvi, Warda Qamar, Bao-Quan Fu, Hong-Bin Yan, Wan-Zhong Jia

**Affiliations:** ^1^State Key Laboratory of Veterinary Etiological Biology, National Professional Laboratory for Animal Echinococcosis, Key Laboratory of Veterinary Parasitology of Gansu Province, Lanzhou Veterinary Research Institute, Chinese Academy of Agricultural Sciences, Lanzhou, China; ^2^Department of Clinical Medicine and Surgery, University of Agriculture, Faisalabad, Pakistan; ^3^Depeartment of Veterinary Medicine, University of Veterinary and Animal Sciences, Lahore, Pakistan; ^4^Department of Medicine, Cholistan University of Veterinary and Animal Sciences, Bahawalpur, Pakistan; ^5^Institute of Pharmacy, Physiology and Pharmacology, University of Agriculture, Faisalabad, Pakistan; ^6^Department of Pathobiology, University of Veterinary and Animal Sciences, Lahore (Jhang Campus), Jhang, Pakistan

**Keywords:** *Coenurus cerebralis*, *cox*1, *Taenia multiceps*, genetic diversity, phylogeny, Pakistan

## Abstract

*Coenurus cerebralis* is the larval stage of *Taenia multiceps* commonly found in the brain (cerebral form), intramuscular and subcutaneous tissues (non-cerebral form) of ungulates. Globally, few reports exist on the molecular characterization and genetic diversity of *C*. *cerebralis* with none available for Pakistan. The current study molecularly characterized 12 *C*. *cerebralis* isolates surgically recovered from sheep (*n* = 4) and goats (*n* = 8) from a total of 3,040 small ruminants using a portion of the cytochrome *c* oxidase subunit 1 (*cox*1) mitochondrial (*mt*) gene. NCBI BLAST search confirmed the identity of each isolate. A high haplotype and a low nucleotide diversity with three haplotypes from the 12 isolates were observed. The findings suggest the existence of unique haplotypes of *C. cerebralis* in Pakistan. The negative value of Tajima's *D* and the positive value of Fu's Fs were inconsistent with population expansion, however, the sample size was small. Bayesian phylogeny revealed that all Pakistani isolates alongside the Chinese sequences (obtained from GenBank) constituted a cluster while sequences from other regions constituted another cluster. This is the first molecular study to determine the genetic diversity of *C*. *cerebralis* in Pakistan and serves as a foundation for prospective studies on the prevalence and population structure of *C. cerebralis* in the country. Furthermore, in this study, we amplified only a partial segment of the *cox*1 gene from a limited sample size. This could have implications on the interpretation of the actual population structure in reality. Thus, we recommend future studies to consider a larger sample size in a massive epidemiological survey for further insights.

## Introduction

The genus *Taenia* is a diverse group of helminths with more than 40 known species that infect livestock and humans causing cysticercosis and/or taeniasis (1, 2). *Coenurus cerebralis* refers to the larval stage of *T. multiceps* (an obligate intestinal tapeworm of dogs) infecting sheep and goat population across the world especially in tropical countries in Asia, Africa, and the Middle East (3). Sheep and goats are the common intermediate hosts but infection has also been reported in cattle and humans (4). Intermediate hosts including sheep and goats often become infected following feeding on vegetation and water contaminated by eggs from dogs (5, 6). This is followed by the development of the larval stage in different tissues including the brain, muscles, and skin (7, 8). The parasite completes its life cycle when the definitive hosts consume poorly discarded infected carcasses, (9). Predominantly, *Coenurus* cysts in the brain (10) present as either acute or chronic form. Acute clinical manifestations include fever, muscle tremors, hemorrhagic retinal lesions, ataxia, blindness, nystagmus, dysmetria, and scoliosis (11, 12). In chronic stages, in addition to acute signs, painful response to pressure over the cystic area, paralysis, blindness, lack of coordination, lethargy, and lack of response to stimuli are evident (13–15). Cyst formation is also frequently seen in subcutaneous and intramuscular tissues (16–21) where they possibly impair normal organ functioning and induce muscular pain (4).

Coenurosis is more prevalent in developing countries (22) with ovine coenurosis varying between 1 and 40% across countries in Africa, Asia, and the Middle East. In Asia and the Middle East, the prevalence in Bangladesh, India, and Jordan has been reported to be 2.5, 2.88, and 3% (13, 23, 24) while in Iran, prevalence in sheep and goat sits between 1.7 and 18.7% (16, 25–28). In countries like Ethiopia, Mozambique, Tanzania, Kenya, and Egypt, the prevalence may range from 1.5 to 42.1% (29–33). In the developed world like the United Kingdom, the infection is uncommon. However, abattoir-based surveys conducted during the 1980s put the prevalence between 0.5 and 5.58% (9). Low prevalence has also been reported in countries like France and Italy (34). Although the infection is cosmopolitan, to the best of our knowledge, only two necropsy-based case reports are available for Pakistan (35, 36).

Genetic variability among many species found within the family taeniidae has been well-documented especially for the genus *Echinococcus*. Nonetheless, the importance and degree of intraspecific variations within some species of *Taenia* remain to be fully understood. However, a good understanding of the genetic diversity and population structure of cestodes remains an important aspect that is essential for the implementation of control and prevention programs as it provides insights related to host specificity or geographical peculiarities. For *T. multiceps*, the genetic variability is poorly studied and there are arguments whether genetic variation may exist between isolates from different hosts, geographical location, or cerebral or non-cerebral forms. In a recent study, the phylogenetic analysis of *nad*1, *cox*1, and 12S rRNA mtDNA sequences showed that no monophyletic groups were based on geographical origin, organ location in the intermediate host (cerebral or non-cerebral), or species of the intermediate host (37). Meanwhile, from Sardinia sheep in Italy, Varcasia et al. (38) reported three variants of *C*. *cerebralis* using the *cox*1 and *nad*1 genes. In Iran, a high scale genetic diversity was reported in *C. cerebralis* isolates obtained from sheep and goats (12, 39) with the *cox*1 gene demonstrating 11 segregation sites resulting in seven haplotypes in sheep (39). Five distinct haplotypes of *C. cerebralis* cysts recovered from sheep, goats and cattle based on partial *cox*1 gene have also been documented in Greece (40). In China, the *cox*1, *cyt*b, *nad*1, and *nad*4 genes have been employed in investigating the genetic diversity of *C. cerebralis* in Sichuan, Gansu, and Inner Mongolia (41–44) and the evidence suggests very low sequence variation 0–0.8%. Li et al. (41) further demonstrated that the *cox*1 mitochondrial gene of *C. cerebralis* obtained from sheep and goats from Gansu province of China were highly conserved.

On the other hand, small ruminant farming is one of the major determinants of advancement in developing countries. However, infectious diseases including parasitic infections have been known to constitute major obstacles to livestock production. Parasitic diseases cause enormous financial losses to owners of sheep and goat population as a result of poor growth, morbidity, poor carcasses value and mortality (34, 45). Mortality in infected sheep and goats reaches about 5% in Ethiopia (32), 25% in Istanbul, Turkey (46) while in Iran losses amounting to 10.2 million rials (27) due to condemnation of infected carcasses highlights the economic setbacks by tapeworm metacestodes. While Pakistan hosts a very large population of small ruminants, little is known on the prevalence, molecular and phylogenetic characteristics of *C*. *cerebralis* infecting small ruminants. Therefore, the current study was designed to molecularly identify and determine the genetic variability of *C. cerebralis* isolates surgically removed from sheep and goats using the *mt* cytochrome oxidase subunit 1 (*cox*1) gene to provide preliminary data for Pakistan besides contributing to our understanding of the global molecular epidemiology and population structure of *C. cerebralis*.

## Materials and Methods

### Study Area

Faisalabad is one of the three most populous cities in Pakistan. Geographically, the city is located 186 meters above sea level lying between 73° and 74° East (longitude), and 30° and 31.5° North (latitude). An extensive canal system and the presence of Ravi and Chenab rivers in the outskirts make the agricultural land highly fertile, fetching the attention of peasants toward agriculture farming and livestock rearing to earn their livelihoods.

### Parasite Material

A total of 3,040 sheep and goats presented to the outdoor patient department of Clinical Medicine and Surgery, University of Agriculture, Faisalabad, Pakistan (irrespective of their disease or purpose of visiting) during May-December, 2019 were palpated thoroughly using digital palpation. Sheep and goats having subcutaneous or intramuscular palpable (cystic) swelling or bulge giving asymmetry to the infested organ were subjected to ultrasound scan procedure for the diagnosis of non-cerebral coenurosis. Hair from the bulging area was clipped and shaved and then animals (sheep and goats) were anesthetized with a combination of xylazine and diazepam at a dose rate of 1 mg/kg (47) and 0.25 mg/kg (48), respectively. Further, local infiltration of 3 ml of 2% lignocaine was also administered subcutaneously. Sterile Aquasonic® 100 Ultrasound Gel (Parker Laboratories) was applied and scanning was performed using 2–5 MHz convex probe (transducer) that was directly placed in the transverse plane over the cystic area using Convex scanner HS-1500 (HONDA® Electronics) and then was spun/moved in cranial and caudal planes for more enhanced visualization of the coenurus cysts. Transcutaneous ultrasonographic confirmation of cysts using convex array was carried out in <1 min followed by ultrasound-guided aseptic aspiration of the cystic fluid using fine needle aspiration technique (FNA). Each cyst was surgically removed and placed carefully in sterile falcon tubes and transferred to the In-House Preventive Veterinary Medicine Public Health Laboratory of the Department of Clinical Medicine and Surgery, University of Agriculture, Faisalabad, Pakistan. Gross morphological identification of the cysts was carried out as described previously by Soulsby (49), Faruk et al. (50), and Oryan et al. (45). The walls of the cysts were translucent and thin filled with a fluid having water like consistency. Protoscolices were firmly adhered to the germinal layer (45).

### DNA Extraction, Amplification, and Sequencing

Genomic DNA from each cyst was extracted using Qiagen Blood and Tissue Kit (Qiagen, Hilden, Germany). A reaction mixture of 25 μl consisting of Ex *Taq*™ (12.5 μl) from Takara Bio, Kusatsu, Japan, forward (10 pmol) and reverse primers 1(10 pmol), extracted genomic DNA (0.5 μl), and RNAse free water up to the final volume of 25 μl was used to carry amplification of the portion of the *cox*1 gene (1,073 bp) using the forward (5′- CTTTGAGTGCGTGGTTGTTG-3′) and reverse (5′- AGAACCTACAGTGCACACAAT-3′) primers. In place of DNA, RNAse free water was added as a negative control. The PCR program was as follows: an initial denaturation at 95°C for 5 min followed by 35 cycles of denaturation (95°C for 30 sec), annealing (55°C for 40 sec), and extension (72°C for 1 min) and a final extension step at 72°C for 10 min. The PCR products were observed under UV light transilluminator using an agarose gel (1.5% w/v) stained with GelRed™. The sizes of the amplicons were estimated by running a 2,000-bp ladder in each gel. The PCR products were sent to Tsingke Biotechnology Company, Beijing for sequencing.

### Molecular Analysis

DNA sequences were corrected manually for any misread nucleotides using Unipro UGENE v1.32.0 software. Using the same software, multiple sequence alignment was carried out. The identities of isolates were confirmed by NCBI BLAST program (https://blast.ncbi.nlm.nih.gov/Blast.cgi).

### Population Genetics and Phylogenetic Analyses

DnaSP 4.5 software was used to calculate nucleotide diversity (π), number of haplotypes (h), and haplotype diversity (Hd). Fu's Fs (51) and Tajima's D (52) were estimated by Arlequin software (53). A median-joining network (54) was constructed based on the partial *mt* cox1 sequences to examine intraspecific variation between haplotypes.

A dataset consisting of *cox*1 gene (639 bp) sequences of the representative haplotypes and other *Taenia* spp. (*T*. *asiatica, T*. *crassiceps, T*. *hydatigena, T. ovis, T*. *pisiformis, T*. *saginata, T*. *solium*, and *T*. *taeniaeformis*) retrieved from GenBank were used to construct a phylogenetic tree using the Bayesian method in MrBayes v.3.1.1 software. *Echinococcus granulosus* whose larvae can infect sheep and goats was used as an out-group.

## Results

### Ultrasonographic Findings

**Figure 1:**
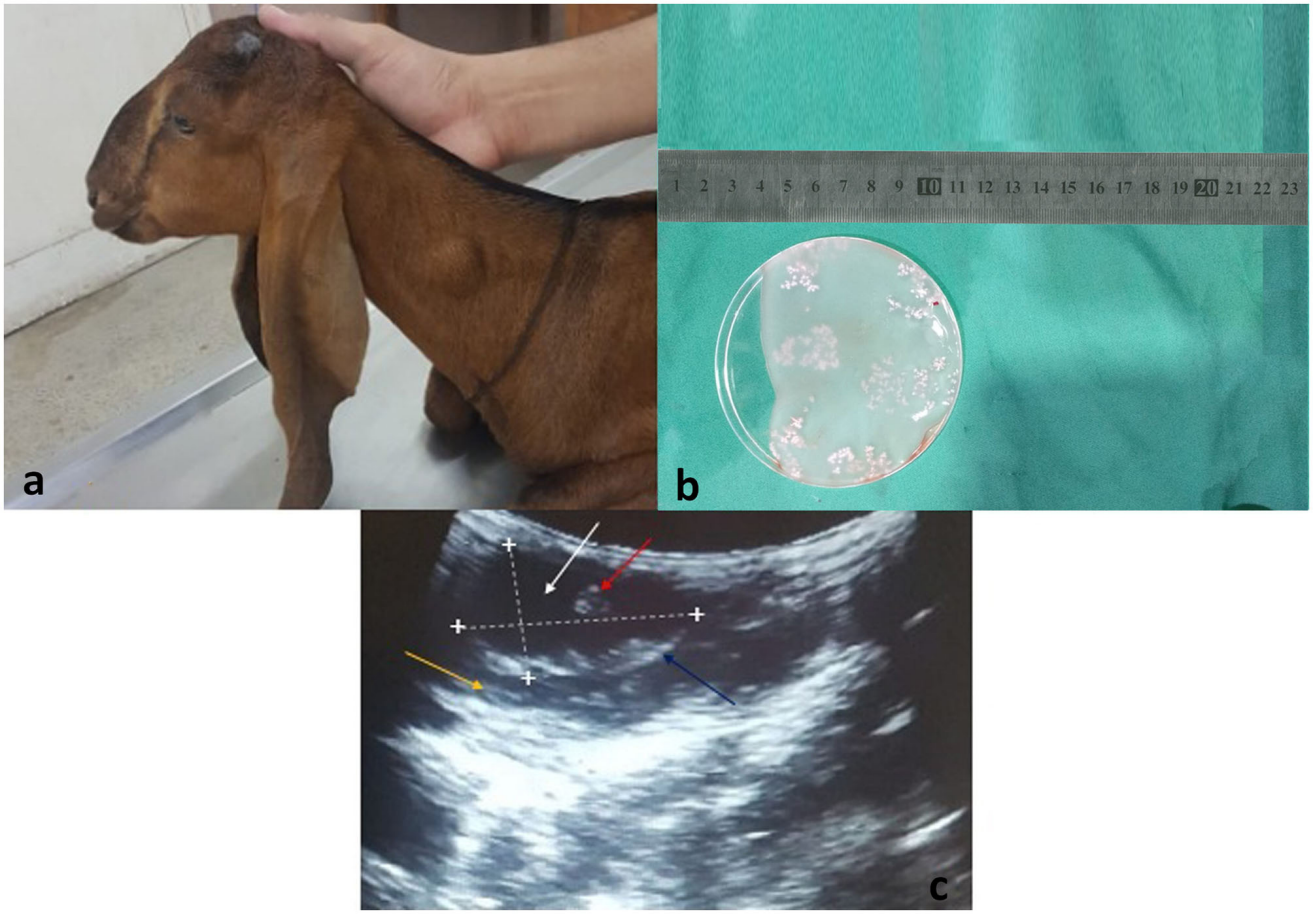
**(a)** A goat presented to the CMS Department of University of Agriculture, Faisalabad, Pakistan with a swollen mass around the neck region. **(b)** Surgically isolated *Coenurus cerebralis* cyst from the neck region of a goat. Scolices are attached to the internal layer of the cyst (white clusters). **(c)** Ultrasound scan of *T*. *multiceps* cyst in goat. Lengthwise and widthwise boundaries of the cyst are demarcated with dotted lines and plus (+) signs. The yellow arrow indicates the host's muscular layer, blue arrow indicates hypoechoic cyst wall, white arrow indicates anechoic cystic fluid, and red arrow indicates protoscolices.

During the study period (May–December, 2019), 4 sheep and 8 goats were found infected with *T*. *multiceps*. The cysts were observed in the face, neck, trunk, and groin regions (Figure 1a). All cysts showed characteristics morphology containing clear fluid of varying volume with many protoscolices. Many scolices were attached to the internal wall of the cyst (Figure 1b). Ultrasound examination showed a hypoechoic cyst wall (Figure 1c, blue arrow) with anechoic cystic fluid (Figure 1c, white arrow) and protoscolices (Figure 1c, red arrow). The cysts appeared as hypoechoic structures with acoustic enhancement at the distal edge.

### Nucleotide Polymorphism and Population Indices

**Figure 2:**
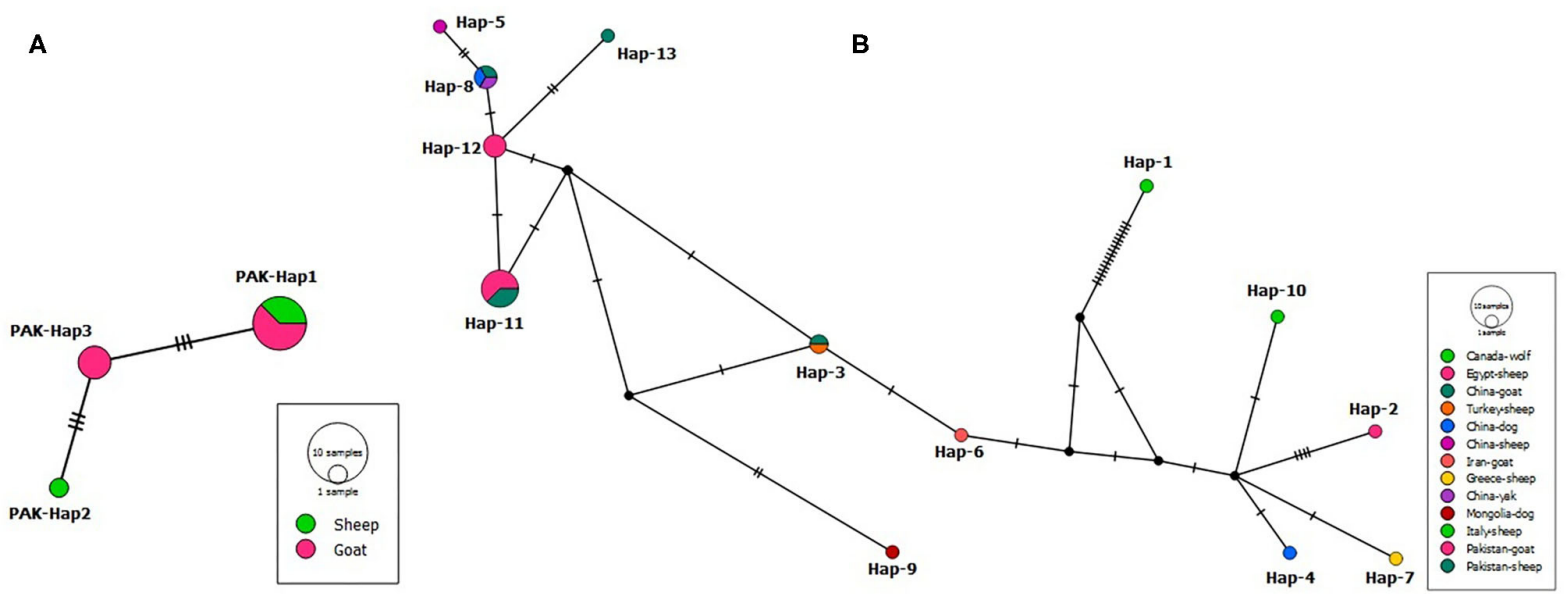
**(A)** Median-joining network of *Coenurus cerebralis* isolates from Pakistani sheep and goats based on *cox*1 gene (639 bp) sequences. Circle sizes are proportional to the haplotype frequencies. Hatch marks represent the number of mutations. Small black spheres are median vectors i.e., unsampled or hypothetical haplotypes. **(B)** Median-joining network of *Taenia multiceps* isolates from different hosts and geographical locations based on the mitochondrial *cox*1 gene (339 bp) sequences. Circle sizes are proportional to the haplotype frequencies. Hatch marks represent the number of mutations. Small black circles are median vectors (i.e., hypothetical haplotypes).

A PCR product of ~1,073 bp was obtained. After multiple sequence alignment, polymorphism analysis resulted in three parsimony informative sites from six mutation sites producing three haplotypes. According to the median-joining network of the partial *cox*1 gene sequences, PAK-Hap3 haplotype comprised only goat isolates, PAK-Hap2 of sheep origin, while PAK-Hap-1 from both hosts (Figure 2A). The examined nucleotide polymorphism resulted in amino acid changes (PAK-Hap2: 103L-103S; PAK-Hap1: 145N-145D).

A nucleotide diversity of 0.00297 and haplotype diversity of 0.718 were observed for the overall *T. multiceps* population. The overall values of Tajima's *D* (−0.06198) and Fu's Fs (2.248) were not significant (*p* > 0.05). A similar observation was also found for *T. multiceps* from the separate populations (sheep and goats) (Table 1).

**Table 1:** Diversity and neutrality indices for *Taenia multiceps* populations from Pakistan.

**Indices**	***cox*****1 (639 bp)**		
	**Goat**	**Sheep**	**Overall**
No. of isolates	8	4	12
No. of mutations	3	6	6
Parsimony informative sites	3	1	3
No. of haplotypes	2	2	3
Haplotype diversity (Hd)	0.730	0.450	0.718
Nucleotide diversity (π)	0.00244	0.00455	0.00297
Tajima's *D*	1.60077	−0.80861	−0.06198
Fu's Fs	2.988	2.944	2.248

To evaluate the geographical relatedness of the 12 Pakistani isolates from this study with those from different parts of the globe, a dataset of 25 *cox*1 sequences (339 bp) was used to draw a median-joining network (Figure 2B). The analysis showed 13 haplotypes, with a high haplotype diversity (0.883), and nucleotide diversity (0.23691). Non-significant Tajima's *D* = 2.24407 and Fu's Fs = 14.002 were obtained.

### Haplotype Data Availability

Sequences of the representative haplotypes from this study have been deposited in the GenBank database under the accession numbers MT863708–MT863710.

### Phylogenetic Analysis

**Figure 3:**
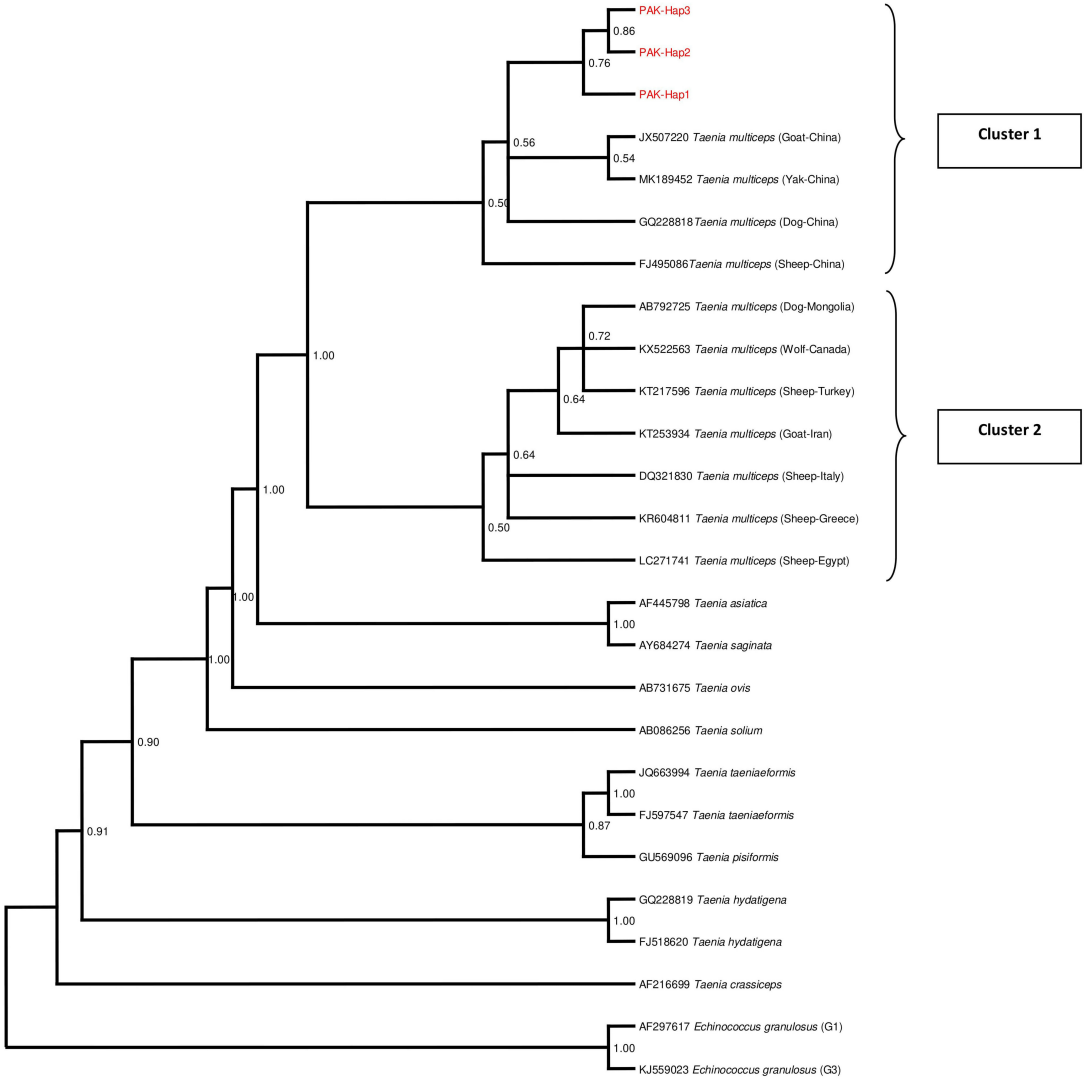
Bayesian phylogeny of Pakistani *Coenurus cerebralis* isolates inferred from the *cox*1 gene. *Echinococcus granulosus* is used as an outgroup. Red = *Coenurus cerebralis* haplotypes representing isolates from this study. Posterior probability values are depicted at the nodes.

The phylogenetic tree was inferred based on the *mt cox*1 gene sequences of the representative haplotypes along with those retrieved from GenBank. The Pakistani and Chinese isolates formed a cluster and were found at a reasonable distance to another cluster comprising isolates from other countries (Figure 3). In the phylogenetic tree, other *Taenia* species (*T*. *asiatica, T*. *crassiceps, T*. *hydatigena, T. ovis, T*. *pisiformis, T*. *saginata, T*. *solium*, and *T*. *taeniaeformis*) were represented while *Echinococcus granulosus sensu stricto* (genotype 1,3) were used as outgroups.

## Discussion

Progress in the caprine and ovine rearing sector is thought to be an important indicator of protracted advancement of developing countries. Parasitic diseases pose a serious threat to livestock production all over the world leading to hampered economic progress (55, 56). Coenurosis is one of the production limiting diseases of ungulates and the exact prevalence is almost impossible to evaluate since the animals are sent for slaughter without prior knowledge of infection by the farmers. Meanwhile, molecular analyses have demonstrated huge importance in the identification and characterization of taeniids cysts (57), as well as the epidemiology, population genetics and phylogeny (58, 59).

Cerebral and non-cerebral coenurosis are terms used to describe infection caused by *T*. *multiceps* based on the location of the coenuri. *T. multiceps* is regarded as the causative agent for cerebral coenurosis while the larval stages of *Multiceps gaigeri* and *M. skrjabini* were previously believed to be responsible for non-cerebral coenurosis in goats and sheep until recently when modern studies have adopted *T. multiceps* to refer to causative agents for both cerebral and non-cerebral coenurosis (37). Information related to non-cerebral coenurosis is scanty and mainly available in countries like Bangladesh (60), India (4, 61, 62), and Iran (63). It has also been reported in Sudan, Oman and Namibia (17).

Here, we detected non-cerebral coenurosis in 12 small ruminants (eight goats and four sheep) out of 3,040 sheep and goats examined from May–December, 2019 which demonstrate a prevalence of 0.39%. Similar low prevalence has been observed in Iran where the prevalence recorded were 0.48 and 1.79% in sheep and goats, respectively (45). A study conducted in Greece to determine the prevalence of non-cerebral coenurosis in sheep also demonstrated a low prevalence of 0.008% (7/90,415) (18).

In this study, a molecular description of *C*. *cerebralis* isolates from sheep and goats was reported for the first time in Pakistan based on *mt cox*1 gene as *mt*DNA remains an important marker in exploring intraspecific variation due to maternal inheritance, conserved structure, higher evolution rate, high genetic divergence, and absence of recombination (64–68). The result demonstrates a high haplotype and low nucleotide diversity comparable to the population indices reported in Italy (Hd = 0.664, π = 0.004) (69) as well as in Egypt, where a considerable number of SNP in the *cox*1 gene, mostly non-parsimony informative with low genetic diversity and fewer haplotypes were reported (70). Although this observation is mostly characteristic of an expanding population, our study revealed inconsistency with population expansion as demonstrated by the population indices: an insignificant negative Tajima's *D* and a positive Fu's Fs, unlike the Italian study where negative but insignificant values were observed for both Tajima's *D* and a positive Fu's Fs (69).

To date, the importance of the nucleotide substitutions and corresponding amino acid changes within *C*. *cerebralis* is unclear (44) though genetic variability leading to phenotypic differences in many other cestodes has been documented (57).

The phylogenetic analysis of the *cox*1 sequences from these isolates revealed a separate cluster suggesting the existence of unique *C*. *cerebralis* haplotypes in Pakistan. This pattern was also observed in the population network and it is also similar to the cluster pattern in a study conducted in Greece (40). All the sequences obtained from GenBank were found to be in one cluster irrespective of the country of origin, predilection site and host while Pakistani isolates occupied a second cluster with the Chinese sequences. This network pattern was also observed by Rostami et al. (22, 39) and Al-Riyami et al. (40) unlike Varcasia et al. (69). Nonetheless, this clustering pattern may indicate a genetic distinction between cerebral and non-cerebral coenurosis agents or intermediate host-specific specificity. Howbeit, further investigation is still warranted. The phylogenetic tree also confirmed *T*. *asiatica* and *T*. *saginata* as the closest to *T. multiceps* as reported previously (41).

## Conclusion

In this study, *C*. *cerebralis* surgically isolated from sheep and goats in Faisalabad, Pakistan were characterized in what we believe is the first attempt to molecularly identify *T*. *multiceps* in Pakistan. Despite the sample size limitation, this study constitutes a significant preliminary data for Pakistan and also a contribution to understanding the global molecular epidemiology and population structure of *C. cerebralis*.

## Data Availability Statement

Representative haplotypes sequences of *cox*1 gene from this study have been deposited in the GenBank database under the accession numbers MT863708–MT863710.

## Ethics Statement

The animal study was reviewed and approved by the Laboratory of Veterinary Preventive Medicine and Public Health, University of Agriculture, Faisalabad, Pakistan. Written informed consent was obtained from the owners for the participation of their animals in this study.

## Author Contributions

MA, H-BY, MS, and W-ZJ conceived and designed the experiments. MT, AAq, AH, and MZ collected the samples. MA, AAl, WQ, and LL carried out the experiments as well as the data analyses. The initial draft of the manuscript was written by MA. JO, AAq, and H-BY. B-QF and W-ZJ gave constructive suggestions for revisions. All authors read and approved the final manuscript.

## Conflict of Interest

The authors declare that the research was conducted in the absence of any commercial or financial relationships that could be construed as a potential conflict of interest.
